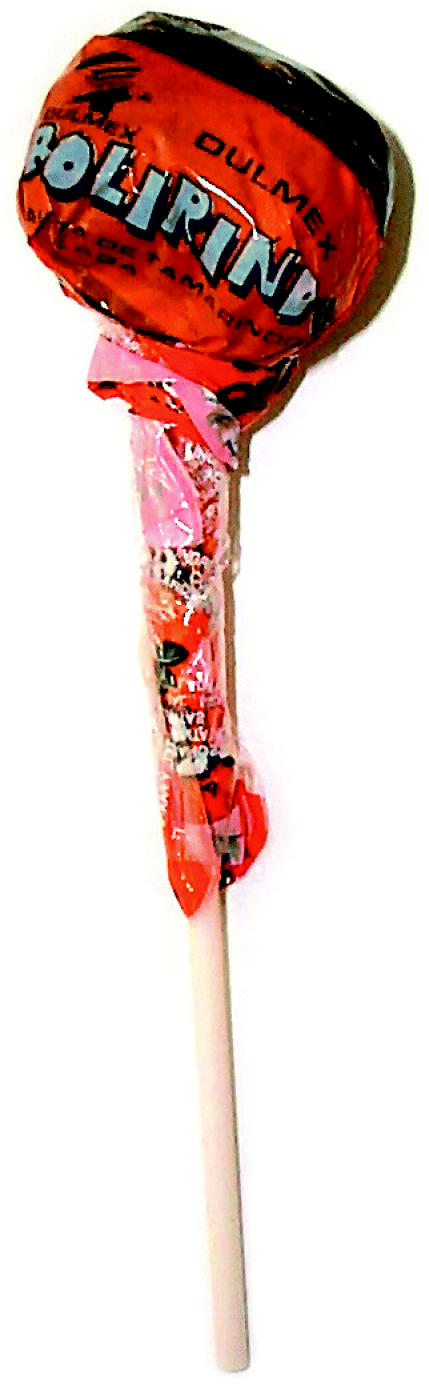# Lead: Sweet Candy, Bitter Poison

**DOI:** 10.1289/ehp.112-a803a

**Published:** 2004-10

**Authors:** Jennifer Medlin

For years, scientists and health experts have traced child lead exposures to paint, leaded gasoline, soils, dust, lead-soldered cans and water pipes, and lead-glazed pottery. More recently, tests have shown that something as presumably innocuous as candies—specifically certain ones made in Mexico—may also be a source of toxic lead. Efforts are under way to combat this source of exposure, but success to date has varied.

Lead poisoning causes a host of problems, many worse in children, including decreased intelligence, impaired neurobehavioral development, stunted physical growth, hearing impairment, and kidney problems. Blood lead levels as low as 10 micrograms per deciliter (μg/dL) are known to cause adverse health effects. The U.S. Food and Drug Administration (FDA) recommends that children under age 6 consume less than 6.0 μg lead daily from all food sources.

Since the early 1990s, the FDA, the California Department of Health Services, and independent laboratories have shown that certain Mexican candies contain sometimes hazardous levels of lead. Historically, some Mexican candy manufacturers have had two versions of their product lines: a cleaner version that meets FDA standards and is designed for export to the United States, and a dirtier—and cheaper—version for the Mexican market. The latter is imported via the “grey market”—pickup trucks that drive shipments over the border to small, family-owned stores, particularly in California, Texas, New Mexico, and Arizona. These stores serve local Latinos, who enjoy the comfort and familiarity of candy from home.

The huge number of these small, informal shipments makes surveillance very difficult. Yet according to the San Diego–based nonprofit Environmental Health Coalition, California has traced 15% of its child lead poisoning cases to lead-contaminated candy—roughly the same percentage attributed to lead-based paint.

Lead-glazed clay pots and candy wrappers printed with lead-based ink were the focus of the FDA’s earliest tests. Tamarind candy packaged in lead-glazed pots elevated blood lead levels in some children to 40–50 μg/dL. And in May 2001, the FDA cautioned consumers to stay away from tamarind Bolirindo lollipops. Tests had found lead concentrations of 21,000 parts per million (ppm) in the lollipops’ wrappers.

California and FDA officials have also found lead in a common ingredient in many Mexican candies—chili powder. Several potential contamination sources have been suggested: soil residue from fields, air-drying or storage where the chilies can accumulate dust from exhaust emissions, metal particles accumulated during the grinding process, and drying over open petrochemical fires. One chili-coated candy tested by the FDA, Chaca Chaca, contained as much as 0.3–0.4 μg lead per gram of product, with one piece of candy alone weighing 35 grams.

Public health and advocacy groups have had some success in warning consumers about lead-contaminated candy, and other efforts to solve the problem are under way. In July 2004, California attorney general Bill Lockyer sued Mexican candy manufacturers and distributors, including two subsidiaries of the Virginia-based Mars candy company, for neglecting to advise consumers that many of their products contain lead. A statement issued by the defendants’ counsel downplayed the extent and gravity of the problem, insisting the companies complied fully with U.S. and international food safety laws. “Traces of minerals such as lead are . . . found in virtually all foods, including fish, meats, grains, fruits and vegetables, and candy,” the statement read. “People have been eating those foods safely for generations.”

Earlier in the summer, California assemblyman Marco Firebaugh proposed a bill that would have banned candies containing more than 0.2 ppm. But the bill died on the floor in August, in large part because other law makers felt the 0.2-ppm safety margin could undermine the Lockyer lawsuit.

The FDA continues to work with the Mexican government to identify the agricultural and manufacturing practices that cause the contamination, although Mexico largely does not have the analytical support, laboratories, and instrumentation to carry out the necessary product testing. Because this contamination appears to be avoidable, the FDA is proposing to lower the amount of lead allowed in candy products and ingredients in the near future.

## Figures and Tables

**Figure:**